# Genetic tools for studying cochlear inhibition

**DOI:** 10.3389/fncel.2024.1372948

**Published:** 2024-03-15

**Authors:** Eleftheria Slika, Paul Albert Fuchs

**Affiliations:** The Center for Hearing and Balance, Otolaryngology-Head and Neck Surgery, Johns Hopkins, University School of Medicine Baltimore, Baltimore, MD, United States

**Keywords:** cochlea, efferent, hair cell, trauma, synaptopathy, gene therapy

## Abstract

Efferent feedback to the mammalian cochlea includes cholinergic medial olivocochlear neurons (MOCs) that release ACh to hyperpolarize and shunt the voltage change that drives electromotility of outer hair cells (OHCs). Via brainstem connectivity, MOCs are activated by sound in a frequency- and intensity-dependent manner, thereby reducing the amplification of cochlear vibration provided by OHC electromotility. Among other roles, this efferent feedback protects the cochlea from acoustic trauma. Lesion studies, as well as a variety of genetic mouse models, support the hypothesis of efferent protection from acoustic trauma. Genetic knockout and gain-of-function knockin of the unique α9α10-containing nicotinic acetylcholine receptor (nAChR) in hair cells show that acoustic protection correlates with the efficacy of cholinergic inhibition of OHCs. This protective effect was replicated by viral transduction of the gain-of-function α9L9’T nAChR into α9-knockout mice. Continued progress with “efferent gene therapy” will require a reliable method for visualizing nAChR expression in cochlear hair cells. To that end, mice expressing HA-tagged α9 or α10 nAChRs were generated using CRISPR technology. This progress will facilitate continued study of the hair cell nAChR as a therapeutic target to prevent hearing loss and potentially to ameliorate associated pathologies such as hyperacusis.

## Introduction

Molecular therapy for inner ear disease is gaining traction through gene replacement for monogenic deafness, as well as small molecule therapies to ameliorate metabolic or ototoxic damage (Lustig and Akil, 2019; Ma et al., 2019). Confounding any therapeutic approach is the continued susceptibility to acoustic overexposure, which can further weaken hair cells and neuronal contacts. Thus, an intriguing strategy is the complementary enhancement of olivocochlear inhibition to minimize acoustic damage. Acoustic protection via cholinergic inhibition of cochlear outer hair cells has been well established by lesion experiments and genetic manipulation in animals but remains to be determined in humans where such methods are not possible (Fuente, 2015). Two strategies have been proposed based on the unique nicotinic AChR (nAChR) of the hair cell: small molecules that can serve as positive allosteric modulators (Elgoyhen et al., 2009) and genetic alteration of the nicotinic AChR of the hair cell (Taranda et al., 2009; Boero et al., 2018, 2020). This mini-review will describe recent advances to facilitate the study of cochlear nAChRs. The ultimate therapeutic goal is not “gene rescue” in the usual sense, but rather the addition of a gain-of-function receptor variant to enhance the native neuronal mechanism for stronger acoustic protection. An appealing aspect of this approach is that olivocochlear efferent neurons are themselves driven by sound in a frequency- and intensity-dependent manner so that the therapeutic effect will be matched to the nature of the threat.

The inner ear is innervated by afferent and efferent neurons that comprise a negative feedback loop (Spoendlin, 1985). In the mammalian cochlea, myelinated type I afferents are excited by glutamate release from inner hair cells (IHCs) to provide all aspects of acoustic sensitivity to the brain (Meyer and Moser, 2010). Sparser, unmyelinated, acoustically insensitive type II afferents ramify among outer hair cells. This was described by Brown (1987) and has been reviewed in Zhang and Coate (2017). Medial olivocochlear efferents (MOCs), driven by afferent connections through the brainstem, release acetylcholine (ACh) to inhibit outer hair cells (OHCs) (Guinan, 1996), while lateral olivocochlear efferents (LOCs) contact type I afferent dendrites, producing a mix of excitation and inhibition (Reijntjes and Pyott, 2016). Inhibition of OHCs by MOCs reduces electromechanical amplification of cochlear vibration, causing maximal loss of sensitivity for IHCs to type I cochlear afferent signaling at the characteristic frequency. This mechanism has been reviewed in Guinan (2010) and Fuchs and Lauer (2019). The frequency- and intensity-dependent acoustic excitation of MOC efferents (Robertson and Gummer, 1985; Liberman and Brown, 1986) thus provides cochlear gain control that is tuned to the acoustic environment. MOC inhibition shifts the dynamic range of afferents and may improve the detection of signals in noise, temporal resolution, and aspects of selective attention, reviewed in Guinan (2010) and Fuchs and Lauer (2019). While definitive evidence for these roles in signal processing is limited, there is agreement that efferent feedback can protect the inner ear from acoustic trauma. This has been shown by lesion studies reviewed in Fuente (2015) and electrical stimulation of MOC efferents (Rajan and Johnstone, 1988; Rajan, 2001).

## Genetically altered mice for studying efferent inhibition

**Figure 1:**
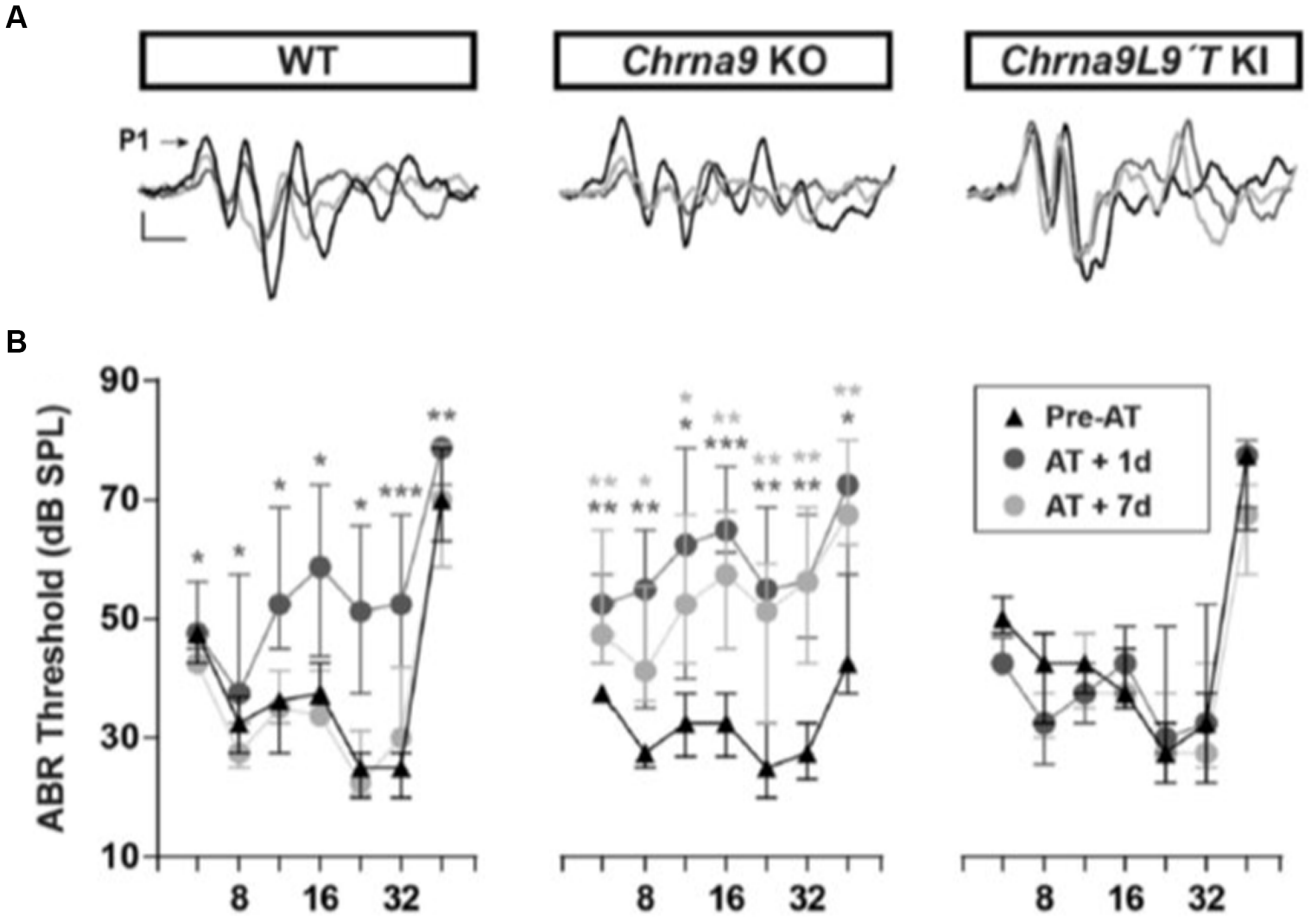
ABR measurements before and after acoustic trauma (AT). **(A)** Representative ABR traces from WT, Chrna9 KO, and Chrna9L9T KI mice at P21 before trauma (Pre-AT, black trace), 1 day after AT (AT1d, dark gray trace), and AT7d (light gray trace). The arrow indicates peak 1 amplitude. Calibration: vertical, 0.4 μV; horizontal, 1 ms. **(B)** ABR thresholds in WT (*n* = 12), Chrna9 KO (*n* = 14), and Chrna9L9T KI (*n* = 15) mice at P21 before AT, 1 day after AT, and 7 days after AT. Median and interquartile ranges are shown, and the comparisons were made using the Friedman tests followed by a *post-hoc* test. Dark gray asterisks represent the statistical significance of AT +1d values compared with Pre-AT, and light gray asterisks represent AT +7d values compared with Pre-AT controls. *p < 0.05; **p < 0.01; ***p < 0.001 (Boero et al., 2018; Figures 2A,B; https://www.jneurosci.org/content/38/34/7440).

The discovery of the genes encoding the subunits of the hair cell nAChR, α9, and α10 (Elgoyhen et al., 1994, 2001) led to the development of mouse models in which these subunits could be knocked out, demonstrating their essential roles (Vetter et al., 2007) and making these animals more prone to acoustic trauma (Lauer and May, 2011; Maison et al., 2013). Equally informative was the subsequent production of point mutation, gain-of-function hair cell nAChR mice (α9L9’T), in which efferent inhibition was greatly enhanced, and noise-induced threshold shifts in ABR and DPOAE were substantially reduced (Taranda et al., 2009). Complementary loss and gain-of-function mouse models have since been used to show that after identical acoustic overexposure, threshold shifts (ABR and DPOAE) are greater in the α9-knockout mice than in wildtype littermates, while the nAChR gain-of-function mice suffered no hearing loss due to these measures (Figure 1; Boero et al., 2018). These studies revealed a similar outcome for measures of noise-induced afferent denervation of IHCs, “synaptopathy.” The amplitude of ABR wave 1 (a measure of the number of afferents responding to a saturating loud sound) was reduced after noise exposure in wildtype mice and α9 knockouts but unchanged compared to pre-exposure magnitude in the α9L9’T gain-of-function transgenic mice.

Commensurate with the changes in ABR wave 1 amplitude, wildtype, and α9-knockout mice lost IHC synapses (paired CtBP2 and GluA2 immunolabel) 7 days after noise damage. Remarkably, α9 gain-of-function IHCs had a small but significant *increase* in the number of IHC ribbon synapses in all cochlear regions compared to controls (average ABR wave 1 amplitude also was larger, but not statistically significant).

A reduction of age-related hearing loss (presbycusis) was demonstrated by comparison of ABR and DPOAE thresholds in mice 6 and 12 months old (Boero et al., 2020). These were elevated 15 dB on average in wildtype mice but unchanged in the α9 gain-of-function mice. Similarly, ABR wave 1 amplitude diminished from 6 to 12 months in wildtype mice but was unchanged in α9 transgenic gain-of-function mice. The α9 gain-of-function mice also had more IHC ribbon synapses at 12 months of age than did the wildtype littermates. Thus, enhanced efferent feedback mitigated both OHC- and IHC-specific pathologies.

## nAChR viral transduction in the mouse cochlea

The correlation between α9 nAChR function and acoustic protection in the genetically modified mice supports the hair cell nAChR as a target to prevent hearing loss in humans. First, however, as for any gene therapy, a number of barriers must be overcome to establish feasibility, reproducibility, and safety. How will the gene product be delivered? Is it expressed at significant levels and localized appropriately? How long does it persist? To begin to address these questions, a series of experiments were carried out using viral carriers to express α9 nAChR subunits in the mouse cochlea. The first foray introduced α9L9’T to “rescue” α9-knockout mice (Zhang et al., 2023), with the aim of replicating the marked differences in acoustic protection observed between knockout and knockin mice (Figure 1).

The modified AAV2.7 m8 (Dalkara et al., 2013) was shown previously to drive widespread expression of green fluorescent protein (GFP) in hair cells and supporting cells of the mouse cochlea (Isgrig et al., 2019). Thus, this virus was constructed commercially to carry the mouse α9L9’T nAChR into the inner ear of homozygous α9-knockout mice (C57BL/6 J genetic background) at postnatal day 0–2 (Zhang et al., 2023). A posterior semi-circular canal approach was used to inject 1–2 μL of virus at ~10^13^ viral copies per ml. Two to three weeks later, the virally produced α9-containing nAChRs were visualized by labeling with Cy3-conjugated RgIA5727, a modified peptide isolated from cone snail venom that binds selectively to α9-containing nAChRs of hair cells (Fisher et al., 2021). Cy3-RgIA5727 puncta were found on the synaptic pole of the majority of OHCs examined at 3 weeks post-injection.

**Figure 2:**
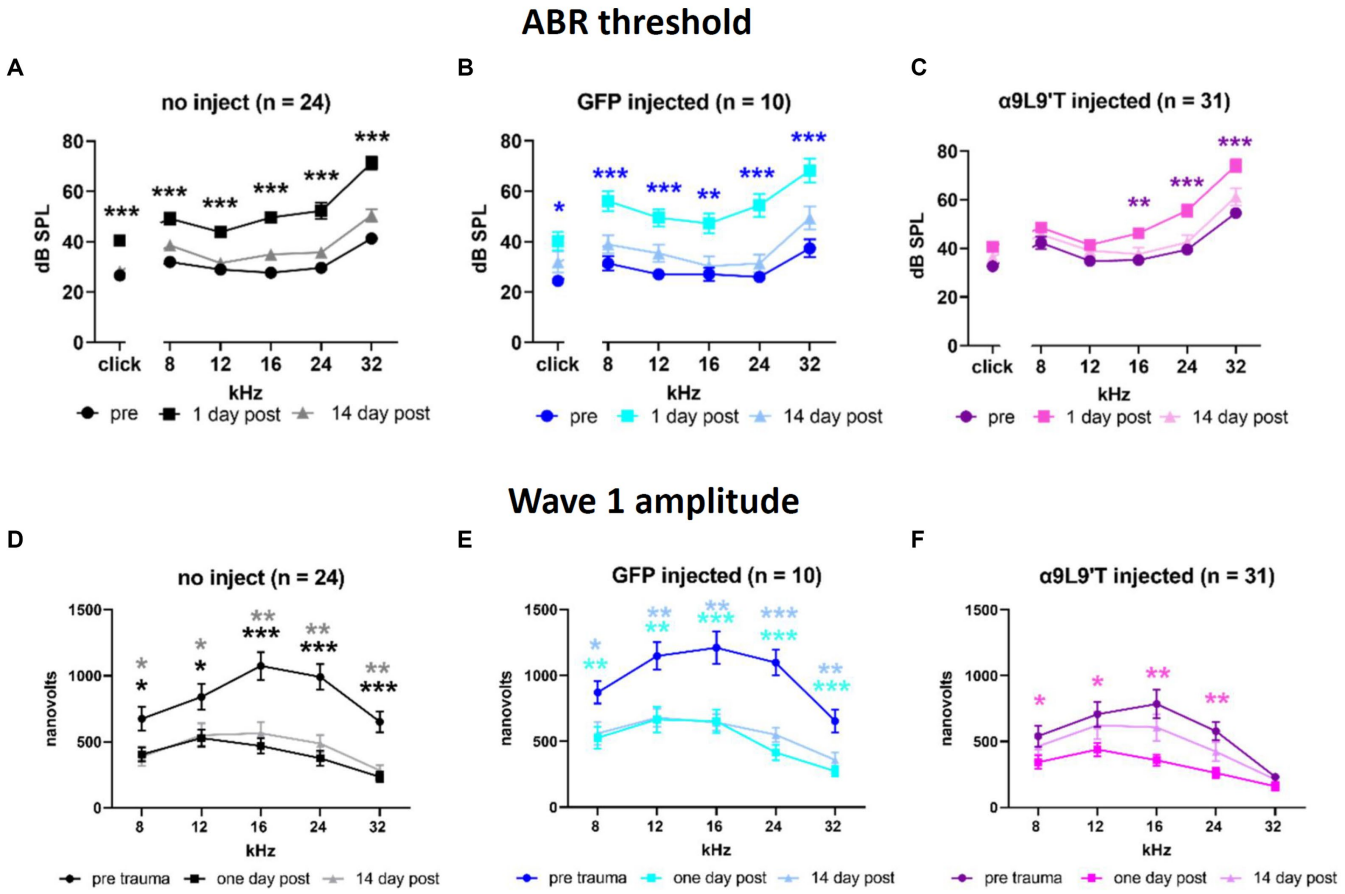
Effect of noise exposure on **(A)** uninjected C67BL/6 J mice, **(B)** mice injected with virus-bearing GFP, and **(C)** mice injected with the α9L9’T-bearing virus ABR thresholds collected prior to 1 and 14 days after acoustic trauma for all cohorts. The amplitude of wave 1 for saturating loud click and tone ABRs for **(D)** uninjected, **(E)** GFP-injected, and **(F)** α9L9’T-injected mice (same cohorts as in upper panels). Darker asterisks denote pre- to 1-day post. Lighter asterisks denote pre- to 14 days post. No significant differences between pre- and 14-day post in α9L9’T-injected mice were observed **(F)**. Statistical significance from multiple unpaired *t*-tests with Welch correction, *p < 0.05, **p < 0.01, ***p < 0.001 [Reprinted with permission from Zhang et al., 2023].

Cohorts of control and experimental mice had hearing tested at 3 weeks of age (ABR thresholds), then exposed to loud sound (2 h @90 dB, 2–20 kHz), and re-tested 1 and 14 days later (clicks and pure tones at 8, 12, 16, 24, and 32 kHz). α9L9’T-injected mice were compared to littermates injected with a virus expressing green fluorescent protein (GFP) and to uninjected littermates (Figure 2). The acoustic trauma protocol caused equivalent upward shifts for click and pure-tone thresholds (hearing loss) 1 day later in uninjected or GFP-injected mice (22 dB shift averaged across all tones and click), while α9L9’T-injected mice experienced approximately half that average shift (12 dB), significant only for the higher frequencies (16, 24, and 32 kHz). In all cohorts, thresholds returned to normal 14 days after trauma.

In addition to measures of threshold that reflect the function of OHCs, IHCs to afferent signaling were examined by measuring the amplitude of wave 1 of the ABR evoked by saturating, loud clicks and tones. All cohorts experienced a 50% drop in wave 1 amplitude 1 day after acoustic trauma; 14 days later, wave 1 showed no recovery in uninjected and GFP-injected mice, but statistically complete recovery in α9L9’T-injected mice. Thus, both OHC damage (threshold shift) and afferent denervation (synaptopathy) in α9-knockout mice were protected to some degree by viral expression of the gain-of-function α9L9’T nAChR.

## Visualizing nAChRs in hair cells with fluorophore-conjugated conotoxin peptide RgIA

Immunolocalization of nAChRs generally, and in the cochlea particularly, has been hampered by the difficulty of producing robust immunolabeling of the receptor. To circumvent this limitation, viral expression of nAChRs was visualized in α9-null mice using a fluorescently tagged biotoxin. Venom from carnivorous cone snails contains a host of biologically active compounds (Olivera et al., 1991) among them a highly selective and potent α9 antagonist, RgIA (Ellison et al., 2006). This has been chemically modified to conjugate with a Cy3 fluorophore. Cy3-RgIA5727 was shown to retain its blocking ability and to label cochlear hair cells at the location of efferent synapses on older outer or younger inner hair cells (Fisher et al., 2021). This labeling is essentially irreversible, making this a promising tool for identifying α9-containing nAChRs in the wide variety of tissues where they have been proposed to act (Liu et al., 2019; Hone and McIntosh, 2023). One limitation however is that Cy3-RgIA5727 only binds in unfixed tissue. In addition, the Cy3 moiety makes the compound sticky so that densely packed tissues tend to accumulate the label and resist washout (e.g., Kolliker’s organ region of immature cochleas) (Fisher et al., 2021).

## Visualizing HA-tagged nAChRs in CRISPRed mice

While Cy3-RgIA5727 was a boon for studying “rescued” α9-null mice and could be useful to localize α9 nAChRs in other tissues, the ultimate goal is to carry out efferent gene therapy on wild-type mice. As Cy3-RgIA5727 will label nAChRs whether native or of viral origin, another tool is needed. Thus, the CRISPR-Cas9 technique was used to produce mice with an HA tag on either the α9 or the α10 subunit of the hair cell nAChR (Vyas et al., 2020). These α9HA, or α10HA mice had no discernible change in hearing (normal ABR thresholds and waveforms), no obvious vestibular deficits (e.g., circling) and growth and breeding appeared normal. When fixed and processed cochlear tissues of adult mice were examined with fluorescence microscopy, HA immunolabel was aligned with SV2-immunolabeled efferent terminals of outer hair cells throughout the cochleas of both α9HA and α10HA mice. Labeling was equivalent in hetero- and homozygotes. In early postnatal mice (P7-P8) when inner hair cells have inhibitory cholinergic synapses, HA immunolabel of inner hair cells was juxtaposed to ChAT-immunolabeled efferent processes, though not at older ages (P20) when the efferent synapses have retracted. In addition to the dense synaptic location of the HA immunolabel, lower level, diffuse cytoplasmic immunoreactivity occurred in young inner hair cells and near the cuticular plate in older outer hair cells. Type II vestibular hair cells also express α9-containing nAChRs (Yu et al., 2020). An ongoing study is examining the distribution of HA-tagged α9-containing nAChRs in these CRISPRed mouse lines, as well as following viral injection of HA-tagged α9L9’T in wild-type mice.

The 9 amino acid HA peptide and an 11 or 12 amino acid spacer were attached to the carboxy tail of either subunit (after transmembrane region 4). This location is predicted to be extracellular, so potentially could interact with other segments, particularly the longer extracellular ligand-binding amino-terminal. To examine the possibility of functional changes, tight-seal whole-cell recordings were made from inner and outer hair cells from apical segments of cochleas from P9 to P11 aged mice (efferent innervation is present on both populations of hair cells at this time and place). Heterozygous and homozygous α9HA and α10HA mice were studied. Electrical stimulation evoked synaptic release while hair cell membrane potential was altered to determine the ionic constituents of the postsynaptic current. In addition, the probability of synaptic release was measured during these long 1-Hz shock trains (that do not cause facilitation) (Ballestero et al., 2011). In all cases, postsynaptic currents included calcium-dependent potassium current as well as cation current through the nAChR, replicating the well-established inhibitory mechanism. Synaptic transmission to inner and outer hair cells of heterozygous α9HA and α10HA mice was quantitatively indistinguishable from that of wild type. However, in homozygous mice (both α9HA and α10HA), the probability of release was significantly lower onto outer hair cells than in heterozygotes (and wildtype). Perhaps related to this, efferent synaptic terminals onto the outer hair cells of homozygous mice (α9HA and α10HA) were significantly smaller (although equal in number) than those onto the outer hair cells of heterozygous mice (Supplementary material in Vyas et al., 2020). It is not certain how presynaptic release efficacy could be altered by HA-tagged postsynaptic receptors, although retrograde facilitation has been observed at these synapses (Kong et al., 2013), perhaps pointing to a change in nAChR binding or gating efficiency. Whatever the cause, future functional studies should employ heterozygous HA mice that have normal synaptic transmission and robust synaptic immunolabeling. The more diffuse cytoplasmic HA labeling also recommends caution if seeking HA-nAChR expression in other tissues. The preferential synaptic localization of the HA label in hair cells and its developmental regulation confirm the biological reality of this expression in cochlear hair cells. Some confirmatory evidence should be sought for other, novel expression patterns.

## Discussion

Substantial progress has been made in detailing the morphology, neurochemistry, and cellular physiology of hair cell inhibition. The efferent projection to the inner ear was identified by Rasmussen in the early 20th century (Rasmussen, 1946). Details of that innervation, including the identity of acetylcholine as a principal neurotransmitter, have been well documented and reviewed in Klinke and Galley (1974). Galambos showed that electrical stimulation of the efferent axons reduced the amplitude of the acoustically evoked compound action potential (Galambos, 1956), while Wiederhold and Kiang confirmed this effect at the level of single cochlear afferents (Wiederhold and Kiang, 1970). Intracellular recordings by Russell in fish (Flock and Russell, 1973, 1976) and frogs (Ashmore and Russell, 1982) provided the first direct evidence for hair cell hyperpolarization by efferent input. This was elaborated by studies in the turtle inner ear that detailed effects on acoustic sensitivity and tuning (Art et al., 1982, 1985; Art et al., 1984; Art and Fettiplace, 1984). Voltage-clamp recording from single isolated chicken hair cells revealed a two-channel mechanism for cholinergic inhibition (Fuchs and Murrow, 1992a,b): calcium influx through a ligand-gated nAChR that drives a far larger increase in calcium-dependent potassium current. This two-channel mechanism of cholinergic inhibition appears to be universal for vertebrate hair cells whether in the cochlea, vestibule, or lateral line. Also universal, efferent terminals are aligned with a near-membrane postsynaptic cistern (Smith and Sjostrand, 1961; Saito, 1980; Fuchs et al., 2014) that may be integral to postsynaptic calcium homeostasis (Lioudyno et al., 2004; Fuchs, 2014; Zachary et al., 2018; Moglie et al., 2021).

The two-channel hypothesis was cemented by the discovery of novel nicotinic receptor subunits, α9 and α10 that comprise the hair cell nAChR (Elgoyhen et al., 1994, 2001). These are distantly related to the muscle and neuronal alpha subunits but differ pharmacologically. In particular, nicotine inhibits, rather than activates, and the most potent small molecule antagonist is strychnine (Elgoyhen et al., 2009). Knockout and gain-of-function knock-in mice have since demonstrated a strong correlation between the function of the hair cell nAChR, and protection from acoustic trauma (Taranda et al., 2009; Boero et al., 2018, 2020). Thus, the ability of efferent feedback to protect from acoustic trauma is well established in animal models, although the significance of this effect for humans remains unsettled. Standard techniques for quantifying efferent feedback, contralateral sound to suppress DPOAEs, show smaller effects in human trials than in animal experiments (Collet et al., 1990; Chambers et al., 2012), consistent with less dense efferent innervation in the human cochlea (Liberman and Liberman, 2019). Nonetheless, the experimental evidence from animals is sufficiently strong to consider the hair cell nAChR as a therapeutic target for the prevention of noise-induced hearing loss, particularly for those at risk of early-onset age-related hearing loss in the military, workplace, or other loud sound environments. Indeed, enhanced efferent function and expanded innervation driven by the gain-of-function nAChR (Murthy et al., 2009; Boero et al., 2018) could have an outsized impact in humans by increasing the modest efferent innervation that declines with age (Liberman and Liberman, 2019). Viral transfection in the mouse cochlea can persist for at least 1 year (Bankoti et al., 2021). Ongoing gene therapy trials (e.g., otoferlin; Qi et al., 2024) will determine this for humans.

An unresolved issue concerns the complex development of efferent innervation of the cochlea. In the first two postnatal weeks in rodents, IHCs express α9-containing nAChRs and are inhibited by ACh release from efferent neurons (Glowatzki and Fuchs, 2000; Simmons, 2002). This transient innervation of IHCs is thought to be important in modulating ribbon synapse maturation and spontaneous afferent firing that shapes central connectivity, reviewed in Rutherford et al. (2021), Frank and Goodrich (2018), and Di Guilmi et al. (2019); 2 weeks postnatally, those IHCs synapses are lost. In contrast, efferent contacts on OHCs begin to function late in the first postnatal week, beginning in the cochlear base and extending to the apex in the second postnatal week (Rohmann et al., 2015), consistent with the basal-to-apical maturation of OHC function (Beurg et al., 2018; Jeng et al., 2020). Thus, both IHCs and OHCs of genetically modified mice could be impacted by altered expression of α9-containing nAChRs. However, it takes 2–3 weeks post-injection for maximal viral expression (Isgrig et al., 2019; Zhang et al., 2023), so early postnatal injection of the gain-of-function α9L9’T may not affect IHC development. Nonetheless, improving the efficacy of viral delivery in adult animals (Zhu et al., 2021) will eliminate development as a confounding factor and will expand future clinical applications.

A second consideration is whether viral transduction will be effective after synaptic maturation is complete. Viral injections in early postnatal mice may benefit by integration of introduced α9 subunits into still-developing synapses. It is conceivable that integration will be suppressed in stabilized adult synapses. However, adult nAChRs do turn over. At the mature neuromuscular junction, bungarotoxin-labeled nAChRs have a half-life of 6–8 days, which is considerably shorter (~2 days) in developing or denervated muscle (Berg and Hall, 1975; Pumplin and Fambrough, 1982; Salpeter and Harris, 1983). This motivates continued study of the pattern and lifetime of viral expression in adult cochleas.

Viral constructs utilized to date employ a strong generic promoter. While useful for the present experiments, such robust expression may not be the best therapeutic strategy. A previous study on the neuronal gain-of-function nAChRs described excitotoxicity due to increased calcium loads (Drenan and Lester, 2012). While this does not happen to hair cells in the knockin mouse lines where expression is under native promoter control, it is conceivable that expression under the strong viral promoter could be disadvantageous. Even in the α9L9’T-knockin mice, there were some unexpected changes. Efferent terminals on OHCs of the α9L9’T mice had reduced quantum content (compensated by increased facilitation ratios) compared to wild-type synapses (Wedemeyer et al., 2018). This could be a beneficial homeostatic adaptation, but other viral constructs, and more extensive studies, including discriminative hearing tasks, are needed to further the ultimate goal of therapeutic translation. For example, OHC-targeted gene therapy with cell-specific promoters could limit off-target effects or overexpression. Additional promise is offered by epigenetic modulation to increase viral transduction (Layman et al., 2015; Chen et al., 2016; Deng et al., 2019).

## Author contributions

ES: Data curation, Formal analysis, Investigation, Methodology, Visualization, Writing – original draft, Writing – review & editing. PF: Conceptualization, Formal analysis, Funding acquisition, Project administration, Resources, Supervision, Visualization, Writing – original draft, Writing – review & editing.

## References

[ref1] Art J. J. Crawford A. C. Fettiplace R. Fuchs P. A. (1982). Efferent regulation of hair cells in the turtle cochlea. Proc. R. Soc. Lond. B Biol. Sci. 216, 377–384. PMID: 6129635 10.1098/rspb.1982.0081

[ref2] Art J. J. Crawford A. C. Fettiplace R. Fuchs P. A. (1985). Efferent modulation of hair cell tuning in the cochlea of the turtle. J. Physiol. 360, 397–421. doi: 10.1113/jphysiol.1985.sp015624, PMID: 3989721 PMC1193468

[ref3] Art J. J. Fettiplace R. (1984). Efferent desensitization of auditory nerve fibre responses in the cochlea of the turtle *Pseudemys scripta elegans*. J. Physiol. 356, 507–523. doi: 10.1113/jphysiol.1984.sp015480, PMID: 6520796 PMC1193179

[ref4] Art J. J. Fettiplace R. Fuchs P. A. (1984). Synaptic hyperpolarization and inhibition of turtle cochlear hair cells. J. Physiol. 356, 525–550. doi: 10.1113/jphysiol.1984.sp015481, PMID: 6097676 PMC1193180

[ref5] Ashmore J. F. Russell I. J. (1982). Effect of electrical stimulation on hair cells of the frog sacculus. J. Physiol. 329:25.

[ref6] Ballestero J. de San Martin J. Z. Goutman J. Elgoyhen A. B. Fuchs P. A. Katz E. (2011). Short-term synaptic plasticity regulates the level of olivocochlear inhibition to auditory hair cells. J. Neurosci. 31, 14763–14774. doi: 10.1523/JNEUROSCI.6788-10.2011, PMID: 21994392 PMC3224807

[ref7] Bankoti K. Generotti C. Hwa T. Wang L. O'Malley B. W. Jr. Li D. (2021). Advances and challenges in adeno-associated viral inner-ear gene therapy for sensorineural hearing loss. Mol Ther Methods Clin Dev 21, 209–236. doi: 10.1016/j.omtm.2021.03.005, PMID: 33850952 PMC8010215

[ref8] Berg D. K. Hall Z. W. (1975). Loss of alpha-bungarotoxin from junctional and extrajunctional a receptors in rat diaphragm muscle *in vivo* and in organ culture. J. Physiol. 252, 771–789. doi: 10.1113/jphysiol.1975.sp011169, PMID: 1206575 PMC1348494

[ref9] Beurg M. Cui R. Goldring A. C. Ebrahim S. Fettiplace R. Kachar B. (2018). Variable number of TMC1-dependent mechanotransducer channels underlie tonotopic conductance gradients in the cochlea. Nat. Commun. 9:2185. doi: 10.1038/s41467-018-04589-8, PMID: 29872055 PMC5988745

[ref10] Boero L. E. Castagna V. C. Di Guilmi M. N. Goutman J. D. Elgoyhen A. B. Gomez-Casati M. E. (2018). Enhancement of the medial Olivocochlear system prevents hidden hearing loss. J. Neurosci. 38, 7440–7451. doi: 10.1523/JNEUROSCI.0363-18.2018, PMID: 30030403 PMC6104299

[ref11] Boero L. E. Castagna V. C. Terreros G. Moglie M. J. Silva S. Maass J. C.. (2020). Preventing presbycusis in mice with enhanced medial olivocochlear feedback. Proc. Natl. Acad. Sci. USA 117, 11811–11819. doi: 10.1073/pnas.2000760117, PMID: 32393641 PMC7261056

[ref12] Brown M. C. (1987). Morphology of labeled afferent fibers in the guinea pig cochlea. J. Comp. Neurol. 260, 591–604. doi: 10.1002/cne.902600411, PMID: 3611412

[ref13] Chambers A. R. Hancock K. E. Maison S. F. Liberman M. C. Polley D. B. (2012). Sound-evoked olivocochlear activation in unanesthetized mice. J. Assoc. Res. Otolaryngol. 13, 209–217. doi: 10.1007/s10162-011-0306-z, PMID: 22160753 PMC3298614

[ref14] Chen J. Hill K. Sha S. H. (2016). Inhibitors of histone deacetylases attenuate noise-induced hearing loss. J. Assoc. Res. Otolaryngol. 17, 289–302. doi: 10.1007/s10162-016-0567-7, PMID: 27095478 PMC4940287

[ref15] Collet L. Kemp D. T. Veuillet E. Duclaux R. Moulin A. Morgon A. (1990). Effect of contralateral auditory stimuli on active cochlear micro-mechanical properties in human subjects. Hear. Res. 43, 251–261. doi: 10.1016/0378-5955(90)90232-E, PMID: 2312416

[ref16] Dalkara D. Byrne L. C. Klimczak R. R. Visel M. Yin L. Merigan W. H.. (2013). *In vivo*-directed evolution of a new adeno-associated virus for therapeutic outer retinal gene delivery from the vitreous. Sci. Transl. Med. 5:189ra76. 10.1126/scitranslmed.3005708 23761039

[ref17] Deng X. Liu Z. Li X. Zhou Y. Hu Z. (2019). Generation of new hair cells by DNA methyltransferase (DNMT) inhibitor 5-azacytidine in a chemically-deafened mouse model. Sci. Rep. 9:7997. doi: 10.1038/s41598-019-44313-0, PMID: 31142766 PMC6541592

[ref18] Di Guilmi M. N. Boero L. E. Castagna V. C. Rodriguez-Contreras A. Wedemeyer C. Gomez-Casati M. E.. (2019). Strengthening of the efferent Olivocochlear system leads to synaptic dysfunction and tonotopy disruption of a central auditory nucleus. J. Neurosci. 39, 7037–7048. doi: 10.1523/JNEUROSCI.2536-18.2019, PMID: 31217330 PMC6733545

[ref19] Drenan R. M. Lester H. A. (2012). Insights into the neurobiology of the nicotinic cholinergic system and nicotine addiction from mice expressing nicotinic receptors harboring gain-of-function mutations. Pharmacol. Rev. 64, 869–879. doi: 10.1124/pr.111.004671, PMID: 22885704 PMC3462994

[ref20] Elgoyhen A. B. Johnson D. S. Boulter J. Vetter D. E. Heinemann S. (1994). Alpha 9: an acetylcholine receptor with novel pharmacological properties expressed in rat cochlear hair cells. Cell 79, 705–715. doi: 10.1016/0092-8674(94)90555-X, PMID: 7954834

[ref21] Elgoyhen A. B. Katz E. Fuchs P. A. (2009). The nicotinic receptor of cochlear hair cells: a possible pharmacotherapeutic target? Biochem. Pharmacol. 78, 712–719. doi: 10.1016/j.bcp.2009.05.023, PMID: 19481062 PMC2737545

[ref22] Elgoyhen A. B. Vetter D. E. Katz E. Rothlin C. V. Heinemann S. F. Boulter J. (2001). alpha10: a determinant of nicotinic cholinergic receptor function in mammalian vestibular and cochlear mechanosensory hair cells. Proc. Natl. Acad. Sci. USA 98, 3501–3506. doi: 10.1073/pnas.051622798, PMID: 11248107 PMC30682

[ref23] Ellison M. Haberlandt C. Gomez-Casati M. E. Watkins M. Elgoyhen A. B. McIntosh J. M.. (2006). Alpha-RgIA: a novel conotoxin that specifically and potently blocks the alpha9alpha10 nAChR. Biochemistry 45, 1511–1517. doi: 10.1021/bi0520129, PMID: 16445293

[ref24] Fisher F. Zhang Y. Vincent P. F. Y. Gajewiak J. Gordon T. J. Glowatzki E.. (2021). Cy3-RgIA-5727 labels and inhibits alpha9-containing nAChRs of Cochlear hair cells. Front. Cell. Neurosci. 15:697560. doi: 10.3389/fncel.2021.697560, PMID: 34385908 PMC8354143

[ref25] Flock A. Russell I. (1973). Efferent nerve fibres: postsynaptic action on hair cells. Nat. New Biol. 243, 89–91. PMID: 4512963

[ref26] Flock A. Russell I. (1976). Inhibition by efferent nerve fibres: action on hair cells and afferent synaptic transmission in the lateral line canal organ of the burbot *Lota lota*. J. Physiol. 257, 45–62. doi: 10.1113/jphysiol.1976.sp011355, PMID: 948076 PMC1309343

[ref27] Frank M. M. Goodrich L. V. (2018). Talking back: development of the olivocochlear efferent system. Wiley Interdiscip. Rev. Dev. Biol. 7:e324. doi: 10.1002/wdev.324, PMID: 29944783 PMC6185769

[ref28] Fuchs P. A. (2014). A 'calcium capacitor' shapes cholinergic inhibition of cochlear hair cells. J. Physiol. 592, 3393–3401. doi: 10.1113/jphysiol.2013.267914, PMID: 24566542 PMC4229337

[ref29] Fuchs P. A. Lauer A. M. (2019). Efferent inhibition of the cochlea. Cold Spring Harb Perspect Med 9:a033530. doi: 10.1101/cshperspect.a033530 30082454 PMC6496333

[ref30] Fuchs P. A. Lehar M. Hiel H. (2014). Ultrastructure of cisternal synapses on outer hair cells of the mouse cochlea. J. Comp. Neurol. 522, 717–729. doi: 10.1002/cne.23478, PMID: 24122766 PMC4474150

[ref31] Fuchs P. A. Murrow B. W. (1992a). Cholinergic inhibition of short (outer) hair cells of the chick's cochlea. J. Neurosci. 12, 800–809. doi: 10.1523/JNEUROSCI.12-03-00800.1992, PMID: 1545240 PMC6576045

[ref32] Fuchs P. A. Murrow B. W. (1992b). A novel cholinergic receptor mediates inhibition of chick cochlear hair cells. Proc. Biol. Sci. 248, 35–40. 1355909 10.1098/rspb.1992.0039

[ref33] Fuente A. (2015). The olivocochlear system and protection from acoustic trauma: a mini literature review. Front. Syst. Neurosci. 9:94. doi: 10.3389/fnsys.2015.00094 26157366 PMC4475794

[ref34] Galambos R. (1956). Suppression of auditory nerve activity by stimulation of efferent fibers to cochlea. J. Neurophysiol. 19, 424–437. doi: 10.1152/jn.1956.19.5.424 13367873

[ref35] Glowatzki E. Fuchs P. A. (2000). Cholinergic synaptic inhibition of inner hair cells in the neonatal mammalian cochlea. Science 288, 2366–2368. doi: 10.1126/science.288.5475.2366, PMID: 10875922

[ref36] Guinan J. J. (1996). “Physiology of olivocochlear efferents” in The cochlea. eds. Dallos P. Arthur Popper N. Richard Fay R. (New York: Springer)

[ref37] Guinan J. J. Jr. (2010). Cochlear efferent innervation and function. Curr. Opin. Otolaryngol. Head Neck Surg. 18, 447–453. doi: 10.1097/MOO.0b013e32833e05d6, PMID: 20717032 PMC3075443

[ref38] Hone A. J. McIntosh J. M. (2023). Nicotinic acetylcholine receptors: therapeutic targets for novel ligands to treat pain and inflammation. Pharmacol. Res. 190:106715. doi: 10.1016/j.phrs.2023.106715, PMID: 36868367 PMC10691827

[ref39] Isgrig K. McDougald D. S. Zhu J. Wang H. J. Bennett J. Chien W. W. (2019). AAV2.7m8 is a powerful viral vector for inner ear gene therapy. Nat. Commun. 10:427. doi: 10.1038/s41467-018-08243-1, PMID: 30683875 PMC6347594

[ref40] Jeng J. Y. Ceriani F. Hendry A. Johnson S. L. Yen P. Simmons D. D.. (2020). Hair cell maturation is differentially regulated along the tonotopic axis of the mammalian cochlea. J. Physiol. 598, 151–170. doi: 10.1113/JP279012, PMID: 31661723 PMC6972525

[ref41] Klinke R. Galley N. (1974). Efferent innervation of vestibular and auditory receptors. Physiol. Rev. 54, 316–357. doi: 10.1152/physrev.1974.54.2.316 4362161

[ref42] Kong J. H. Zachary S. Rohmann K. N. Fuchs P. A. (2013). Retrograde facilitation of efferent synapses on cochlear hair cells. J. Assoc. Res. Otolaryngol. 14, 17–27. doi: 10.1007/s10162-012-0361-0, PMID: 23183877 PMC3540278

[ref43] Lauer A. M. May B. J. (2011). The medial olivocochlear system attenuates the developmental impact of early noise exposure. J. Assoc. Res. Otolaryngol. 12, 329–343. doi: 10.1007/s10162-011-0262-7, PMID: 21347798 PMC3085693

[ref44] Layman W. S. Williams D. M. Dearman J. A. Sauceda M. A. Zuo J. (2015). Histone deacetylase inhibition protects hearing against acute ototoxicity by activating the Nf-kappaB pathway. Cell Death Discov 1:15012. doi: 10.1038/cddiscovery.2015.12, PMID: 26279947 PMC4536828

[ref45] Liberman M. C. Brown M. C. (1986). Physiology and anatomy of single olivocochlear neurons in the cat. Hear. Res. 24, 17–36. doi: 10.1016/0378-5955(86)90003-1, PMID: 3759672

[ref46] Liberman L. D. Liberman M. C. (2019). Cochlear efferent innervation is sparse in humans and decreases with age. J. Neurosci. 39, 9560–9569. doi: 10.1523/JNEUROSCI.3004-18.2019, PMID: 31628179 PMC6880465

[ref47] Lioudyno M. Hiel H. Kong J. H. Katz E. Waldman E. Parameshwaran-Iyer S.. (2004). A "synaptoplasmic cistern" mediates rapid inhibition of cochlear hair cells. J. Neurosci. 24, 11160–11164. doi: 10.1523/JNEUROSCI.3674-04.2004, PMID: 15590932 PMC6730265

[ref48] Liu Q. Li M. Whiteaker P. Shi F. D. Morley B. J. Lukas R. J. (2019). Attenuation in nicotinic acetylcholine receptor alpha9 and alpha10 subunit double Knock-out mice of experimental autoimmune encephalomyelitis. Biomol. Ther. 9:827. doi: 10.3390/biom9120827, PMID: 31817275 PMC6995583

[ref49] Lustig L. Akil O. (2019). Cochlear gene therapy. Cold Spring Harb Perspect Med 9:a033191. doi: 10.1101/cshperspect.a033191 30323014 PMC6719588

[ref50] Ma Y. Wise A. K. Shepherd R. K. Richardson R. T. (2019). New molecular therapies for the treatment of hearing loss. Pharmacol. Ther. 200, 190–209. doi: 10.1016/j.pharmthera.2019.05.003, PMID: 31075354 PMC6626560

[ref51] Maison S. F. Usubuchi H. Liberman M. C. (2013). Efferent feedback minimizes cochlear neuropathy from moderate noise exposure. J. Neurosci. 33, 5542–5552. doi: 10.1523/JNEUROSCI.5027-12.2013, PMID: 23536069 PMC3640841

[ref52] Meyer A. C. Moser T. (2010). Structure and function of cochlear afferent innervation. Curr. Opin. Otolaryngol. Head Neck Surg. 18, 441–446. doi: 10.1097/MOO.0b013e32833e0586 20802334

[ref53] Moglie M. J. Wengier D. L. Elgoyhen A. B. Goutman J. D. (2021). Synaptic contributions to Cochlear outer hair cell ca(2+) dynamics. J. Neurosci. 41, 6812–6821. doi: 10.1523/JNEUROSCI.3008-20.2021, PMID: 34253627 PMC8360681

[ref54] Murthy V. Taranda J. Elgoyhen A. B. Vetter D. E. (2009). Activity of nAChRs containing alpha9 subunits modulates synapse stabilization via bidirectional signaling programs. Dev. Neurobiol. 69, 931–949. doi: 10.1002/dneu.20753, PMID: 19790106 PMC2819290

[ref55] Olivera B. M. Rivier J. Scott J. K. Hillyard D. R. Cruz L. J. (1991). Conotoxins. J. Biol. Chem. 266, 22067–22070. doi: 10.1016/S0021-9258(18)54531-2 1939227

[ref56] Pumplin D. W. Fambrough D. M. (1982). Turnover of acetylcholine receptors in skeletal muscle. Annu. Rev. Physiol. 44, 319–335. doi: 10.1146/annurev.ph.44.030182.001535 7041799

[ref57] Qi J. Tan F. Zhang L. Lu L. Zhang S. Zhai Y.. (2024). AAV-mediated gene therapy restores hearing in patients with DFNB9 deafness. Adv Sci (Weinh):e2306788. doi: 10.1002/advs.202306788 38189623 PMC10953563

[ref58] Rajan R. (2001). Noise priming and the effects of different cochlear centrifugal pathways on loud-sound-induced hearing loss. J. Neurophysiol. 86, 1277–1288. doi: 10.1152/jn.2001.86.3.1277, PMID: 11535676

[ref59] Rajan R. Johnstone B. M. (1988). Binaural acoustic stimulation exercises protective effects at the cochlea that mimic the effects of electrical stimulation of an auditory efferent pathway. Brain Res. 459, 241–255. doi: 10.1016/0006-8993(88)90640-3, PMID: 3179705

[ref60] Rasmussen G. L. (1946). The olivary peduncle and other fiber projections of the superior olivary complex. J. Comp. Neurol. 84, 141–219. doi: 10.1002/cne.900840204, PMID: 20982804

[ref61] Reijntjes D. O. J. Pyott S. J. (2016). The afferent signaling complex: regulation of type I spiral ganglion neuron responses in the auditory periphery. Hear. Res. 336, 1–16. doi: 10.1016/j.heares.2016.03.011, PMID: 27018296

[ref62] Robertson D. Gummer M. (1985). Physiological and morphological characterization of efferent neurones in the guinea pig cochlea. Hear. Res. 20, 63–77. doi: 10.1016/0378-5955(85)90059-0, PMID: 2416730

[ref63] Rohmann K. N. Wersinger E. Braude J. P. Pyott S. J. Fuchs P. A. (2015). Activation of BK and SK channels by efferent synapses on outer hair cells in high-frequency regions of the rodent cochlea. J. Neurosci. 35, 1821–1830. doi: 10.1523/JNEUROSCI.2790-14.2015, PMID: 25653344 PMC4315822

[ref64] Rutherford M. A. von Gersdorff H. Goutman J. D. (2021). Encoding sound in the cochlea: from receptor potential to afferent discharge. J. Physiol. 599, 2527–2557. doi: 10.1113/JP279189, PMID: 33644871 PMC8127127

[ref65] Saito K. (1980). Fine structure of the sensory epithelium of the guinea pig organ of Corti: afferent and efferent synapses of hair cells. J. Ultrastruct. Res. 71, 222–232. doi: 10.1016/S0022-5320(80)90108-2, PMID: 7381992

[ref66] Salpeter M. M. Harris R. (1983). Distribution and turnover rate of acetylcholine receptors throughout the junction folds at a vertebrate neuromuscular junction. J. Cell Biol. 96, 1781–1785. doi: 10.1083/jcb.96.6.1781, PMID: 6853602 PMC2112453

[ref67] Simmons D. D. (2002). Development of the inner ear efferent system across vertebrate species. J. Neurobiol. 53, 228–250. doi: 10.1002/neu.10130, PMID: 12382278

[ref68] Smith C. A. Sjostrand F. S. (1961). Structure of the nerve endings on the external hair cells of the guinea pig cochlea as studied by serial sections. J. Ultrastruct. Res. 5, 523–556. doi: 10.1016/S0022-5320(61)80025-7, PMID: 13914158

[ref69] Spoendlin H. (1985). Anatomy of cochlear innervation. Am. J. Otolaryngol. 6, 453–467. doi: 10.1016/S0196-0709(85)80026-0 3909832

[ref70] Taranda J. Maison S. F. Ballestero J. A. Katz E. Savino J. Vetter D. E.. (2009). A point mutation in the hair cell nicotinic cholinergic receptor prolongs cochlear inhibition and enhances noise protection. PLoS Biol. 7:e18. doi: 10.1371/journal.pbio.1000018 19166271 PMC2628405

[ref71] Vetter D. E. Katz E. Maison S. F. Taranda J. Turcan S. Ballestero J.. (2007). The alpha10 nicotinic acetylcholine receptor subunit is required for normal synaptic function and integrity of the olivocochlear system. Proc. Natl. Acad. Sci. USA 104, 20594–20599. doi: 10.1073/pnas.0708545105, PMID: 18077337 PMC2154476

[ref72] Vyas P. Wood M. B. Zhang Y. Goldring A. C. Chakir F. Z. Fuchs P. A.. (2020). Characterization of HA-tagged alpha9 and alpha10 nAChRs in the mouse cochlea. Sci. Rep. 10:21814. doi: 10.1038/s41598-020-78380-5, PMID: 33311584 PMC7733449

[ref73] Wedemeyer C. Vattino L. G. Moglie M. J. Ballestero J. Maison S. F. Di Guilmi M. N.. (2018). A gain-of-function mutation in the alpha9 nicotinic acetylcholine receptor alters medial Olivocochlear efferent short-term synaptic plasticity. J. Neurosci. 38, 3939–3954. doi: 10.1523/JNEUROSCI.2528-17.2018, PMID: 29572431 PMC5907056

[ref74] Wiederhold M. L. Kiang N. Y. (1970). Effects of electric stimulation of the crossed olivocochlear bundle on single auditory-nerve fibers in the cat. J. Acoust. Soc. Am. 48, 950–965. doi: 10.1121/1.1912234, PMID: 5480390

[ref75] Yu Z. McIntosh J. M. Sadeghi S. G. Glowatzki E. (2020). Efferent synaptic transmission at the vestibular type II hair cell synapse. J. Neurophysiol. 124, 360–374. doi: 10.1152/jn.00143.2020, PMID: 32609559 PMC7500374

[ref76] Zachary S. Nowak N. Vyas P. Bonanni L. Fuchs P. A. (2018). Voltage-gated calcium influx modifies cholinergic inhibition of inner hair cells in the immature rat cochlea. J. Neurosci. 38, 5677–5687. doi: 10.1523/JNEUROSCI.0230-18.2018, PMID: 29789373 PMC6010563

[ref77] Zhang K. D. Coate T. M. (2017). Recent advances in the development and function of type II spiral ganglion neurons in the mammalian inner ear. Semin. Cell Dev. Biol. 65, 80–87. doi: 10.1016/j.semcdb.2016.09.017, PMID: 27760385 PMC5393967

[ref78] Zhang Y. Hiel H. Vincent P. F. Y. Wood M. B. Elgoyhen A. B. Chien W.. (2023). Engineering olivocochlear inhibition to reduce acoustic trauma. Mol Ther Methods Clin Dev 29, 17–31. doi: 10.1016/j.omtm.2023.02.011, PMID: 36941920 PMC10023855

[ref79] Zhu J. Choi J. W. Ishibashi Y. Isgrig K. Grati M. Bennett J.. (2021). Refining surgical techniques for efficient posterior semicircular canal gene delivery in the adult mammalian inner ear with minimal hearing loss. Sci. Rep. 11:18856. doi: 10.1038/s41598-021-98412-y, PMID: 34552193 PMC8458342

